# Association between religious/spiritual coping and quality of life among hemodialysis patients in Ecuador

**DOI:** 10.3389/fpubh.2025.1510329

**Published:** 2025-04-02

**Authors:** Patricia Bonilla Sierra, José Miguel Pérez-Jiménez, Denisse Paulina Espinoza Quezada, Giancarlo Lucchetti, Rocío De-Diego-Cordero

**Affiliations:** ^1^Department of Nursing, Schools of Nursing, Physiotherapy and Podiatry, University of Seville, Seville, Spain; ^2^Department of Health Sciences, Technical Private University of Loja, Loja, Ecuador; ^3^Research Group CTS1149: Comprehensive and Sustainable Health: Bio-Psycho-Social, Cultural and Spiritual Approach to Human Development, Sevilla, Spain; ^4^Anaesthesiology and Resuscitation Clinical Management Unit, University Hospital Virgen Macarena, Sevilla, Spain; ^5^School of Medicine, Federal University of Juiz de Fora, Juiz de Fora, Brazil

**Keywords:** advanced chronic kidney disease, religious coping, quality of life, hemodialysis, spirituality

## Abstract

There is evidence concerning the association between religiousness and quality of life in hemodialysis patients However, studies carried out in South America are scarce in the literature, particularly in Ecuador. This study aims to investigate the influence of religious/spiritual coping on the quality of life of Ecuadorian hemodialysis patients. This is a cross-sectional study carried out in a reference public hospital in Ecuador. Hemodialysis patients answered a questionnaire including sociodemographics, medical data, quality of life (“Kidney Disease Quality of Life-KDQOL-36), SF-12 and religious/spiritual coping (Abbreviated Religious/Spiritual Coping Scale-Brief-RCOPE). Unadjusted and adjusted models were carried out to investigate the association between religious/spiritual coping and quality of life. A total of 58 participants were included. Hemodialysis patients presented higher levels of positive than negative religious/spiritual coping. Although religious/spiritual positive coping was associated with level of education, the relationship between religious/spiritual coping and quality of life were not statistically significant. Despite the lack of statistical significance, our findings highlight the widespread use of R/S coping among hemodialysis patients, emphasizing the need to integrate spiritual support into clinical care. This study provides valuable insights into a predominantly Catholic population (98.3%) in Ecuador, contributing to the limited research on R/S coping in Latin America. Given the homogeneous religious profile, future studies should include more diverse populations and longitudinal designs to assess its impact on quality of life. The lack of a significant association may be influenced by the religious homogeneity of the sample, as well as factors such as social support and illness perception, warranting further exploration in future research.

## Introduction

Chronic kidney disease (CKD) is defined as the presence of kidney damage and/or a decrease in glomerular filtration rate (GFR) for more than 3 months. Although the diagnosis of renal failure is established when the GFR is less than 60 ml/min/1.73 m^2^, when GFR is less than 20 ml/min/1.73 m^2^, replacement therapy should be considered using renal transplantation, peritoneal dialysis or hemodialysis (1, 2).

The most common replacement therapy available for public health systems is hemodialysis. Patients with end-stage renal disease (ESRD) undergoing hemodialysis may experience several stressors that can impact their physical and mental health as well as their quality of life. Treatment conditions and the chronic course of the disease limit these patients and therefore trigger stress, social isolation, limitations in locomotion and walking, decreased physical activities, dependency, and feelings of fear and insecurity about health and well-being (3, 4). There are various ways of coping with these stressors, including self-care, adapting to the characteristics of the disease, socializing, family relationships, and seeking religious or spiritual support (5).

Using religious or spiritual beliefs to cope with the problems faced by life challenges, conflicts, and diseases is commonly referred to as religious/spiritual coping (6, 7). Spirituality and/or religiosity (R/S) are two important elements in coping with a chronic or terminal illness, helping them to cope with their illness as a source of strength and having a positive impact on their physical and emotional symptoms (8–11).).

The role of religiosity and spirituality (R/S) in improving the quality of life and mental health of patients undergoing hemodialysis has been well-documented (12, 13). Studies suggest that R/S serves as an essential coping mechanism for individuals with chronic illnesses such as chronic kidney disease (CKD). Religious practices and spiritual beliefs are associated with higher levels of happiness, reduced feelings of depression, and improved overall mental health in hemodialysis patients. This is likely due to the emotional support, sense of purpose, and social connections often provided by religion and spirituality. Furthermore, studies indicate that R/S contributes to a better perception of quality of life, which is crucial for patients facing the physical and emotional challenges of long-term treatments, such as dialysis (14–16).

Although religiosity and spirituality are often associated with positive health outcomes, some studies have also found that negative religious coping strategies can have detrimental effects on the quality of life and mental health of hemodialysis patients (17). Negative religious coping, characterized by feelings of abandonment due to a higher power or questioning one’s faith, can lead to increased psychological distress, reduced vitality, and impaired social and emotional functioning. Research indicates that patients who utilize negative religious coping strategies experience poorer physical and mental health, including increased anxiety, depression, and diminished overall sense of well-being (18).

Although there is growing evidence for this relationship, most studies are still conducted in developed countries, which include specific cultural backgrounds and have good access to treatment. Therefore, studies focusing on developing countries are essential to better understand the role of religious and spiritual beliefs in contexts with limited resources. In South America, some studies have been conducted in Brazil (19–22), however, to the best of our knowledge, there is a scarcity of research in other countries with different cultural backgrounds and a history of Spanish colonization, such as Ecuador. The burden of kidney disease in Ecuador is significant, with chronic kidney disease (CKD) on the rise largely due to diabetes and hypertension. In 2018, there were approximately 17,484 patients on dialysis, representing 567 per million people, and dialysis treatment accounted for over 11% of the public health budget (23). To address this gap, this study aimed to investigate the influence of religious and spiritual coping on the quality of life of hemodialysis patients in Ecuador.

End-stage renal disease (ESRD) represents a significant public health issue in Ecuador, with a rising prevalence and a substantial impact on the healthcare system. Given that hemodialysis is the predominant treatment for these patients, understanding the factors that influence their quality of life (QoL) is crucial. Religion and spirituality play a significant role in coping with chronic illnesses, particularly in countries with a strong religious tradition such as Ecuador, where approximately 80% of the population identifies as Catholic (22).

However, the literature on the relationship between religious/spiritual (R/S) coping and QoL in ESRD patients in Latin America is limited, with most studies focusing on developed countries with distinct sociocultural contexts. Therefore, this study aims to investigate the association between R/S coping and QoL in Ecuadorian hemodialysis patients, hypothesizing that positive R/S coping is associated with a better perceived quality of life.

This research will contribute to filling a gap in the literature and provide relevant evidence to enhance psychosocial and spiritual support in the management of ESRD in the region.

## Methods

### Study design and setting

This is a cross-sectional study carried out to determine the relationship between spiritual and religious coping and quality of life in patients with advanced chronic kidney disease undergoing hemodialysis treatment at the Isidro Ayora General Hospital in Loja, Ecuador, in the period August 2022–February 2023. Given its cross-sectional design, this study did not allow for causal inferences, limiting the ability to establish cause-and-effect relationships.

The study was conducted in the hemodialysis unit of the Isidro Ayora General Hospital in Loja, a public hospital that provides free care to patients from Zonal Coordination 7, which includes the provinces of El Oro, Loja, and Zamora Chinchipe in the southern region of Ecuador. This unit has 12 hemodialysis machines and serves an average of 64 chronic patients who receive treatment three times per week, with approximately 1,070 dialysis sessions performed monthly. In 2022, 10,800 sessions were conducted for permanent patients, along with an additional 300 sessions for emergencies.

The study included 58 hemodialysis patients, selected from a public referral hospital in southern Ecuador. Although the sample size may be considered small, the Hemodialysis Unit at Isidro Ayora General Hospital serves a fixed and stable population of chronic patients. Additionally, due to the nature of hemodialysis treatment, patients undergo prolonged and exhausting procedures, which limit their availability to participate in extensive interviews. While the sample size does not allow for the generalization of results at a national level, it adequately represents the hemodialysis population in this specific region, with demographic and religious characteristics consistent with the Ecuadorian context.

To ensure the reliability and validity of the collected data, several strategies were implemented to mitigate potential selection biases. Strict inclusion and exclusion criteria were applied to guarantee sample homogeneity. Response bias and social desirability bias were minimized through individualized interviews during hemodialysis, conducted by a trained researcher using validated questionnaires (Brief-RCOPE and KDQOL-36) while ensuring confidentiality of responses. Additionally, the administration of instruments was standardized, and the process was closely monitored to prevent interviewer bias.

These measures ensure the quality and reliability of the data, allowing the findings to contribute to the understanding of religious/spiritual coping in patients with chronic kidney disease in Ecuador, with the potential to be expanded in future studies with larger samples.

### Eligibility criteria

The study included patients over 18 years of age from the hemodialysis service at Isidro Ayora Hospital in Loja diagnosed with advanced chronic kidney disease according to the criteria of the International Society of Nephrology (24). These patients were oriented to time, place, and person, clinically stable, of both sexes, able to understand and respond to the questions, and agreed to participate by signing the informed consent form. The exclusion criteria included those with significant cognitive impairment, severe hearing disabilities, or those who did not provide informed consent.

### Instruments

Several instruments designed to gather comprehensive and accurate information about the participants in key areas were utilized for data collection. The Brief-RCOPE and KDQOL-36 were chosen for their efficiency and relevance to hemodialysis patients. The Brief-RCOPE provides a concise yet comprehensive assessment of positive and negative R/S coping, reducing patient burden compared to longer tools like the full RCOPE. The KDQOL-36, unlike generic QoL scales (e.g., SF-36), includes CKD-specific domains, offering a more accurate evaluation of quality of life in this population. These instruments were selected for their validity, reliability, and suitability, ensuring a thorough yet practical assessment in a clinical setting.

### Sociodemographic data

The sociodemographic data questionnaire was composed of age (years), sex (“Male” or “Female”), marital status (“Single,” “Married,” “Divorced,” or “Widowed”), monthly income (Ecuadorian currency) and religion (“Catholic,” “Evangelical,” “Jehovah’s Witness,” “Church of Jesus Christ of Latter-day Saints,” “Agnostic,” and “Other”).

### Medical data

The questionnaire was designed to gather information regarding the participants’ health status, focusing on their diagnosis of kidney disease, time since diagnosis, duration of hemodialysis treatment (with categories such as <1 year, 1–3 years, 4–5 years, 6–8 years, >8 years), presence of comorbidities (with response options such as 1, 2, 3, 4 or more, or “none reported”), and the number of hemodialysis sessions per week. Response options were tailored to each question to ensure precise and detailed data capture.

### Religious/spiritual coping

To assess how participants coped with their illness from a religious or spiritual perspective, the Brief Religious Coping Scale (Brief-RCOPE) was used, consisting of 14 questions. This scale employs a Likert-type format, in which participants rated their responses on a scale from 1 (never) to 4 (always). The total score was calculated by summing the responses, with a higher score indicating a greater use of positive religious coping. The version was validated in Spanish in an Argentinian context, demonstrating both reliability and validity (25).

### Kidney disease-related quality of life

The “Kidney Disease Quality of Life” (KDQOL-36) instrument was employed, consisting of 36 questions that assess several key dimensions related to the quality of life of patients with kidney disease, including symptoms and problems, effects of kidney disease, burden of kidney disease, and general health aspects. This questionnaire also uses a Likert-type format, with responses scored on a scale from 0 to 100, with a higher score representing a better quality of life. The scale has been validated in Spanish in previous studies, confirming its applicability in Spanish-speaking contexts (26).

### Procedure

A request was issued to the directors of the hospital center chosen to carry out the research. Once approved, the objectives of the research, their rights, autonomy and voluntary participation were explained to the staff of the hemodialysis unit and to the patients on the different days and times assigned.

Informed consent was obtained from the patients, who signed to assure their voluntary participation in the project, knowing that their identity would remain anonymous and that they could withdraw from the project if they felt uncomfortable or did not wish to participate.

During the hemodialysis sessions, the patients were approached for the interview, which was conducted by a trained investigator in the nephrology area. This setting allowed the investigator to interact directly with the patients while they were available, thus facilitating data collection in familiar environments. The questionnaire, which took approximately 10 min to complete, was interviewer-administered, meaning that the investigator read the questions and recorded the responses, allowing for any doubts to be clarified in real-time and ensuring data accuracy. This approach helped ensure that patients fully understood the questions and felt comfortable answering them, thereby guaranteeing the quality and reliability of the collected information.

### Statistical analyses and sample

All patients (*n* = 64) undergoing hemodialysis sessions in the hospital were invited to participate into the study. First, a descriptive analysis using absolute and relative numbers for categorical variables, and means and standard deviations for continuous measures were reported. Subsequently, inferential analyses were performed as follows: First, categorical variables were dichotomized as follows: sex (male vs. female), education (None/Basic vs. HighSchool/University), occupation (not working/retired vs. working), and Marital Status (not married vs. married). The association between sociodemographic and data, Brief-RCOPE, and KDQOL-36 were carried out using independent t-tests and Pearson correlations.

To test the main hypothesis of the study, unadjusted linear regression models were first used with positive and negative spiritual/religious coping as independent variables and the KDQOL-36 and SF-12 dimensions as dependent variables. These models were adjusted according to sociodemographic and medical variables that reached a *p* < 0.20 in the t-tests and Pearson correlations. The variables included in the adjustment were selected based on their potential influence on R/S coping and quality of life, as supported by previous research on hemodialysis patients. Factors such as age, sex, education level, and income were considered due to their known associations with coping mechanisms and psychosocial well-being. Including these variables ensured a more accurate interpretation of the relationships studied, enhancing the robustness of the analytical approach.

All analyses were performed using SPSS version 20, and statistical significance was set at *p* < 0.05. R-square values were provided for the final adjusted regression models. Since some variables did not assume normality, log-transformations (log10) were carried out for the following variables: positive and negative spiritual/religious coping and the KDQOL-36 and SF-12 dimensions.

### Ethical aspects

All participants were informed about the study objectives and were asked to sign a written informed consent of their participation. They were guaranteed anonymity and data confidentiality in the information provided. This study was approved by the Research Committee of the Technical Private University of Loja, Ecuador, under reference number 0045620CTM.

## Results

Of the initial sample of 64 patients, 58 voluntarily participated in the study and 6 were excluded because they did not wish to participate in the study. No patient was excluded for other reasons.

Table 1 presents the participants’sociodemographic and medical characteristics. The sample was composed predominantly of males (65.5%), with a mean age of 57.0 (SD 14.2), a mean of 1.71 (SD 0.87), low education (58.6%), married (65.5%), Catholics (98.3%), Income <US$400 (65.5%), and 3–8 years of disease (53.5%). Table 1 also presents the scores for the instruments used, showing that the use of positive coping was higher than that of negative coping (31.20 vs. 11.50). The religious homogeneity of the sample (98.3% Catholic) limited the ability to explore differences between religious affiliations, which may have reduced variability in responses.

**Table 1:** Sociodemographic characteristics of the sample.

	n	%
Sex		
Male	38	65.5
Female	20	34.5
Education		
No education	2	3.4
Elementary	32	55.2
High School	20	34.5
University	4	6.9
Marital Status		
Single	13	22.4
Widowed	7	12.1
Married	38	65.5
Religion		
Catholic	57	98.3
Evalgelical	1	1.7
Income		
<400	38	65.5
401 to 800	17	29.3
>800	3	5.2
Time of disease		
<1	5	8.6
1 to 3	14	24.1
3 to 5	15	25.9
5 to 8	16	27.6
>8	8	13.8

	Mean	SD
Age	57.00	14.24
Number of Diseases	1.71	0.87
Positive Religious Coping	31.20	3.34
Negative Religious Coping	11.50	3.52
SintomasProblemas	76.22	13.69
EfectosEnfermedad	71.60	17.76
CargaEnfermedad	43.10	30.61
SF12 Physical	33.26	8.03
SF12 Mental	45.00	10.55

Within positive religious coping, the highest scoring question was “I seek God’s closeness,” while the lowest scoring question was “I try to understand that God strengthens me through certain situations.” As for negative religious coping the highest scoring question was “I feel convinced that the devil makes things happen” while the lowest scoring question was “I question God’s love for me.” Positive religious coping revealed a mean of 31.21 points, with the lowest score being 19 and the highest being 35, while negative religious coping revealed a mean of 11.5 points, with the lowest score being 7 and the highest being 25.

Table 2 shows the associations between sociodemographic factors, medical variables and religious/spiritual coping. Positive religious coping was significantly associated (*p* < 0.05) with education, indicating that patients with secondary or higher education had lower scores in negative coping and higher scores in positive coping. It was also marginally associated (*p* < 0.20) with sex and age. In contrast, negative coping was not significantly (*p* < 0.05) associated with any variable but showed a marginally association (*p* < 0.20) with education and income. Although not statistically significant, patients over 60 years old tended to report greater use of positive religious coping (*p* = 0.083), while men showed lower positive coping scores compared to women (*p* = 0.166).

**Table 2:** Association between spiritual/religious coping and sociodemograhic variables.

	Positive coping		Negative coping	
	Mean (SD)^*^	*p*	Mean (SD)^*^	*p*
Sex				
Male	1.48 (0.05)		1.04 (0.12)	
Female	1.50 (0.03)	0.160	1.03 (0.13)	0.657
Education				
None/Basic	1.50 (0.03)		1.06 (0.12)	
HighSchool/University	1.47 (0.06)	0.025	1.00 (0.13)	0.079
Ocupation				
Not working/Retired	1.48 (0.05)		1.05 (0.13)	
Working	1.49 (0.04)	0.820	1.02 (0.12)	0.346
Marital Status				
Not married	1.47 (0.07)		1.04 (0.15)	
Married	1.49 (0.03)	0.213	1.03 (0.11)	0.785

	Pearson Coefficient	*p*	Pearson Coefficient	*p*
Age	0.241	0.069	−0.044	0.746
Education	−0.269	0.041	−0.300	0.022
Income	0.115	0.391	−0.261	0.048
Time of disease	0.044	0.742	−0.122	0.362
Number of diseases	0.031	0.816	−0.027	0.843

* Log-transformed values (log10).

Table 3 presents the association between sociodemographic, medical, and quality of life variables. Symptoms/problems were significantly (*p* < 0.05) related to sex, and marginally (*p* < 0.20) related to education, occupation, and the number of diseases. The effects of disease were not significantly (*p* < 0.05) related to any variable but marginally (*p* < 0.20) related to education. The burden of disease was significantly (*p* < 0.05) related to education and marginally (*p* < 0.20) related to the time of disease. KDQOL-36 Physical Health was significantly (*p* < 0.05) related to age, and marginally (*p* < 0.20) related to occupation and the number of diseases. KDQOL-36 Mental Health was not significantly (*p* < 0.05) related to any variable, but marginally (*p* < 0.20) related to sex and education.

**Table 3:** Association between sociodemographic characteristics and quality of life outcomes.

	Symptoms/Problems		Effects of kidney disease		Burden of kidney disease		SF12 physical		SF 12 mental health	
	Mean (SD)^*^	*p*	Mean (SD)^*^	*p*	Mean (SD)^*^	*p*	Mean (SD)^*^	*p*	Mean (SD)^*^	*p*
Sex										
Male	1.89 (0.07)		1.84 (0.13)		1.54 (0.37)		1.51 (0.10)		1.66 (0.09)	
Female	1.84 (0.09)	0.022	1.83 (0.10)	0.675	1.63 (0.24)	0.369	1.48 (0.11)	0.291	1.60 (0.11)	0.064
Education										
None/Basic	1.85 (0.08)		1.81 (0.12)		1.51 (0.34)		1.52 (0.12)		1.61 (0.11)	
HighSchool/University	1.89 (0.07)	0.100	1.87 (0.11)	0.073	1.65 (0.32)	0.150	1.49 (0.09)	0.457	1.67 (0.09)	0.047
Occupation										
Not working/Retired	1.89 (0.07)		1.81 (0.13)		1.51 (0.37)		1.48 (0.09)		1.63 (0.11)	
Working	1.85 (0.10)	0.121	1.85 (0.10)	0.217	1.63 (0.30)	0 0.207	1.52 (0.12)	0.165	1.64 (0.10)	0.619
Marital Status										
Not married	1.87 (0.08)	0.845	1.84 (0.13)	0.863	1.66 (0.22)	0.224	1.53 (0.09)	0.235	1.64 (0.11)	0.969
Married	1.87 (0.08		1.83 (0.11		1.49 (0.11)		1.49 (0.11)		1.64 (0.10)	

	Pearson Coefficient	*p*	Pearson Coefficient	*p*	Pearson Coefficient	*p*	Pearson Coefficient	*p*	Pearson Coefficient	*p*
Age	−0.077	0.567	−0.025	0.853	−0.006	0.964	−0.334	0.010	−0.050	0.709
Education	0.100	0.454	0.158	0.235	0.194	0.167	0.050	0.708	0.202	0.129
Income	0.140	0.295	0.105	0.434	0.054	0.703	0.124	0.353	0.076	0.569
Time of disease	−0.064	0.632	0.080	0.551	0.326	0.018	−0.060	0.657	−0.022	0.871
Number of diseases	−0.202	0.128	−0.140	0.295	0.069	0.629	−0.202	0.129	−0.094	0.482

* Log-transformed values (log10).

Negative religious coping did not show significant correlations with quality of life in both adjusted and unadjusted models (*p* > 0.05) (Table 3). However, patients with higher levels of negative coping tended to report greater illness burden and lower emotional well-being (*p* = 0.085 and *p* = 0.100, respectively) (Table 4).

**Table 4:** Unadjusted and adjusted models for the relationship between spiritual/religious coping and quality of life outcomes.^*^

	Positive					Negative				
	Unadjusted	*p*	Adjusted	*p*	R-square	Unadjusted	*p*	Adjusted	*p*	R-square
Symptoms/Problems	−0.106	0.427	−0.001^1^	0.995	0.197	−0.014	0.914	0.002^2^	0.986	0.196
Effects of kidney disease	−0.221	0.095	−0.169^3^	0.229	0.102	−0.139	0.297	−0.087^4^	0.534	0.064
Burden of kidney disease	−0.095	0.501	−0.064^5^	0.656	0.165	−0.203	0.148	−0.152^6^	0.297	0.150
SF12 Physical	0.090	0.500	0.168^7^	0.222	0.181	0.068	0.610	0.048^8^	0.735	0.159
SF 12 Mental	−0.133	0.321	−0.031^9^	0.824	0.137	−0.226	0.088	−0.212^10^	0.118	0.154

* Log-transformed values (log10).

1 Sex, education, age, occupation, number diseases.

2 Sex, education, income, occupation, number diseases.

3 Sex, education, age.

4 Education, income.

5 Sex, education, age, time of disease.

6 Education, income, time of disease.

7 Sex, education, age, occupation, time of disease.

8 Education, income, occupation, age, time of disease.

9 Sex, education, age.

10 Education, income, sex.

Table 4 show the linear regression models. There were no statistically significant associations between religious/spiritual coping and quality of life in either the unadjusted and of adjusted models. Suggesting a weak or non-existent relationship in the analyzed sample.

## Discussion

Our findings showed that hemodialysis patients in Ecuador had higher levels of positive than negative religious/spiritual coping. Although religious/spiritual positive coping was associated with level of education level, the relationship between religious/spiritual coping and quality of life was not statistically significant.

Several studies have explored the use of positive religious coping among hemodialysis patients, highlighting the different levels of prevalence and effects on quality of life. Our study found that a high percentage of hemodialysis patients used positive religious coping as a strategy to deal with their illness, similar to the findings reported by Taheri-Kharameh et al. (18), Lucchetti et al. (27), and Yıldırım-Üşenmez et al. (28), who observed a high mean score of positive coping strategies in their Iranian, Brazilian and Turkish patients. All these studies agree that positive religious coping is widely used among hemodialysis patients, although the exact levels of prevalence and impact may vary across different cultural contexts.

Concerning the relationship between religious/spiritual coping strategies and quality of life, previous studies have found that religious coping was associated with health-related quality of life among 170 Brazilian hemodialysis patients (29), with a brief index of quality of life (EQ-5D) among 362 Muslim patients (30), with SF-36 pain and physical and social functioning among 218 Brazilian hemodialysis patients (31) and with overall WHOQOL-Bref among 169 Brazilian hemodialysis patients (32).

Despite this previous evidence, our study failed to detect differences. Although the non-significance of our findings can be related to the small sample size, our adjusted Betas ranged from −0.091 to −0.209, showing that even with larger sample sizes, the associations would remain weak. In this context, it seems that, although Ecuadorian hemodialysis patients used their religious and spiritual beliefs aiming to cope with their problems as noted by the scores of RCOPE, this was not associated with their quality of life. Ecuador is a highly Catholic country (almost 80% of the population) (22), with an indigenous background and a previous conflict between religious freedom and the government. In addition, there is a growing tendency of a change on the religious backgrounds in the younger generation in Ecuador, resulting in an increase of spiritual, but not religious individuals and also of atheists. The sample in this study consisted almost entirely of Catholic patients, reflecting the deep-rooted religious tradition among older generations in Ecuador. With an average age of 57 years (SD = 14), participants tended to maintain a traditional religious perspective, which influenced their religious/spiritual (R/S) coping. Additionally, since the study population comprised individuals with advanced chronic kidney disease, with most being over 44 years old, this means that millennials (Generation Y) were not included. This generation is characterized by a more flexible and personalized spirituality, less adherence to traditional religious structures, and greater openness to practices such as mindfulness, yoga, and holistic well-being (33). These changes could also have an influence on our results and future studies should understand further this relationship.

The results of our study are consistent with other research, in which lower quality of life scores in the physical and mental components among hemodialysis patients. This reflects the burden of illness experienced by patients, particularly in the physical and mental health dimensions, which are often compromised. A study conducted in Ecuador by Méndez et al. (34) identified lower scores in the physical and mental components of the Kidney Disease Quality of Life (KDQOL-36) scale, with the burden of kidney disease dimension showing the most negative results. These findings are replicated in Cuba, where Capote-Leyva et al. (35) found that only the burden of illness dimension was associated with poor quality of life, whereas other dimensions, such as symptoms and problems, were rated as good. Quality of life should be a measure to be taken into account in the assessment and treatment of advanced chronic kidney disease and hemodialysis patients to ensure proper treatment and support for patients throughout their disease process.

To improve the quality of life for hemodialysis patients, it is essential to recognize and integrate religious and spiritual support into clinical care, as these beliefs are considered important to patients. Future interventions should be tailored to demographic factors such as age, gender, and education level, as these seem to influence religious coping strategies. Longitudinal studies are needed to understand how religious coping evolves over time and its long-term impact on health. Additionally, it would be valuable to replicate this study in different cultural contexts to analyze how religious variations influence coping and quality of life. Training healthcare professionals to sensitively address the spiritual needs of patients is crucial, as is investigating negative religious coping, which can have negative psychological consequences. Previous studies have shown that integrating religious/spiritual (R/S) support into clinical care contributes to greater treatment adherence, reduced anxiety, and a stronger therapeutic bond between the patient and the healthcare team (31, 36). Additionally, implementing standardized protocols for assessing R/S coping can help healthcare professionals personalize care according to patients’ beliefs and values, promoting a more holistic approach to the management of chronic kidney disease. However, this study also highlights the need to develop strategies to address negative religious coping, as it may lead to feelings of guilt or hopelessness, negatively affecting patients’ quality of life. Therefore, the integration of R/S support should be structured and evidence-based, taking into account the diversity of beliefs while respecting each patient’s autonomy. Finally, developing spiritual assessment tools will help healthcare professionals identify the specific needs of patients and provide appropriate support throughout their treatment process.

## Limitations

The main limitation of this study is the small sample size, which may be too small to establish a significant relationship. In addition, regarding the nature of the population in terms of religion, the vast majority of the participants are Catholics, may have influenced our results, not allowing to detect differences in religious coping between different religions and quality of life. Finally, the cross-sectional design of this study limited the generalizability of the findings. Larger longitudinal studies are needed to better understand the relationships between religiosity/spirituality and the quality of life of hemodialysis patients in Ecuador.

The main limitation of this study is the small sample size (*n* = 58), which may reduce the statistical power to establish significant associations. However, this sample represents a high participation rate (90.6%) of eligible patients within the studied dialysis unit, providing valuable insights into this population, particularly in a country where “Spirituality and Health” studies are scarce and underexplored. Additionally, the homogeneity of the sample in terms of religion, with the vast majority identifying as Catholic (98.3%), may have influenced the results, limiting the ability to detect differences in religious coping across diverse religious groups. Nevertheless, it is important to highlight that Ecuador is predominantly a Catholic country with little religious diversity, which is in line with our findings. This highlights the need for future studies to include a more religiously diverse population to better assess the variability in coping mechanisms and their impact on quality of life. Furthermore, the cross-sectional design of this study restricts the ability to determine causal relationships between religious/spiritual coping and quality of life. To address these limitations, future research should employ larger, multicenter, and longitudinal studies that allow for a more comprehensive understanding of how religiosity and spirituality influence the well-being of hemodialysis patients over time in Ecuador.

## Conclusion

Our results support that hemodialysis patients use religious and spiritual strategies to cope with the problems associated with their hemodialysis. The use of positive was higher than the negative strategies in our sample, highlighting that most individuals tend to use religion in a functional manner.

An association between quality of life and religious coping was not demonstrated in this study.

Studies with larger numbers of participants assessing the issues of quality of life and spirituality or religious coping in patients with advanced chronic kidney disease should be encourage in Ecuador, aiming to offer and develop better treatment modalities and disease support. A possible connection between religious coping and quality of life should be further investigated, not only in this population but also in all patients with advanced chronic or end-of-life illnesses in all facilities and levels of care.

## Data Availability

The original contributions presented in the study are included in the article/supplementary material, further inquiries can be directed to the corresponding author.

## References

[1] Chen TK Knicely DH Grams ME. Chronic kidney disease diagnosis and management: a review. JAMA. (2019) 322:1294–304. doi: 10.1001/jama.2019.14745, PMID: 31573641 PMC7015670

[2] Vaidya SR Aeddula NR. (2024) Chronic kidney disease. In: StatPearls [Internet]. Treasure Island (FL): Available online at: https://www.ncbi.nlm.nih.gov/books/NBK535404/ (Accessed July 31, 2024).

[3] Almutary H Al-Ghamdi R Miajan Z Alharbi A Badokhon R Alharazi R. Exploring the needs of patients undergoing hemodialysis: a qualitative study. Cureus. (2023) 15:e50076. doi: 10.7759/cureus.50076, PMID: 38192957 PMC10771957

[4] Dembowska E Jaroń A Gabrysz-Trybek E Bladowska J Gacek S Trybek G. Quality of life in patients with end-stage renal disease undergoing hemodialysis. J Clin Med. (2022) 11:1584. doi: 10.3390/jcm11061584, PMID: 35329910 PMC8949549

[5] Zaripov S Turaev I Rakhimov S. Assessment of the quality of life in patients with chronic kidney disease in the practice of hemodialysis. J Modern Educ Achiev. (2023) 6:103–9.

[6] Graça L Brandão T. Religious/spiritual coping, emotion regulation, psychological well-being, and life satisfaction among university students. J Psychol Theol. (2024) 52:342–58. doi: 10.1177/00916471231223920

[7] Ramírez Jiménez MG González-Arratia López-Fuentes NI Barcelata Eguiarte BE. Religious coping and spirituality as mediators between perceived stress and resilience in adults with type 2 diabetes mellitus. LIBERABIT. Revista Peruana De Psicología, (2022) 28:e569. doi: 10.24265/liberabit.2022.v28n2.569

[8] Arrey AE Bilsen J Lacor P Deschepper R. Spirituality/religiosity: a cultural and psychological resource among sub-Saharan African migrant women with HIV/AIDS in Belgium. PLoS One. (2016) 11:e0159488. doi: 10.1371/journal.pone.0159488, PMID: 27447487 PMC4957758

[9] Iannello NM Inguglia C Silletti F Albiero P Cassibba R Lo Coco A. How do religiosity and spirituality associate with health-related outcomes of adolescents with chronic illnesses? A scoping review. Int J Environ Res Public Health. (2022) 19:13172. doi: 10.3390/ijerph192013172, PMID: 36293751 PMC9603522

[10] Korniejczuk V. A. Moroni C. M. Quiyono Escobar E. Rodríguez Gómez J. Valderrama Rincón A. Charles-Marcel Z. L.. (2020). La salud espiritual, su evaluación y su papel en la obesidad, la diabetes mellitus y otras enfermedades crónicas no-transmisibles. Nutrición, obesidad, BDM, HTA, dislipidemias, TCA, salud mental. pp. 561–586.

[11] Vardar O Serçekus P Özkan S. Spirituality and religion on coping with cancer for patients and caregivers. J Educ Res Nurs. (2021) 18:462–6. doi: 10.5152/jern.2021.35651

[12] Brečka TA Ptáček R Sebalo I Anders M Sebalo Vňuková M. Impact of religion and spirituality on the incidence of depression and mental health among young adults in the Czech Republic. Front Psychol. (2024) 15:1423730. doi: 10.3389/fpsyg.2024.1423730, PMID: 39268390 PMC11391637

[13] Rambod M Pasyar N Mokhtarizadeh M. Psychosocial, spiritual, and biomedical predictors of Hope in hemodialysis patients. Int J Nephrol Renov Dis. (2020) 13:163–9. doi: 10.2147/IJNRD.S255045, PMID: 32617015 PMC7326188

[14] Al-Ghabeesh SH Alshraifeen AA Saifan AR Bashayreh IH Alnuaimi KM Masalha HA. Spirituality in the lives of patients with end-stage renal disease: a systematic review. J Relig Health. (2018) 57:2461–77. doi: 10.1007/s10943-018-0622-2, PMID: 29671169

[15] Burlacu A Artene B Nistor I Buju S Jugrin D Mavrichi I. Religiosity, spirituality and quality of life of dialysis patients: a systematic review. Int Urol Nephrol. (2019) 51:839–50. doi: 10.1007/s11255-019-02129-x, PMID: 30919258

[16] Siqueira J Fernandes NM Moreira-Almeida A. Association between religiosity and happiness in patients with chronic kidney disease on hemodialysis. J Bras Nefrol. (2019) 41:22–8. doi: 10.1590/2175-8239-jbn-2018-0096, PMID: 30421782 PMC6534031

[17] Alradaydeh MF Khalil AA. The association of spiritual well-being and depression among patients receiving hemodialysis. Perspect Psychiatr Care. (2018) 54:341–7. doi: 10.1111/ppc.12249, PMID: 29077991

[18] Taheri-Kharameh Z Zamanian H Montazeri A Asgarian A Esbiri R. Negative religious coping, positive religious coping, and quality of life among hemodialysis patients. Nephrourol Monthly. (2016) 8:e38009. doi: 10.5812/numonthly.38009, PMID: 27896237 PMC5120233

[19] Boas GV Nakasu MVP. Association between resilience, religiosity and therapeutic adherence in patients undergoing hemodialysis/Associação entre resiliência, religiosidade e adesão terapêutica em pacientes submetidos a hemodiálise. Rev Med. (2021) 100:119–27. doi: 10.11606/issn.1679-9836.v100i2p119-127

[20] Bravin AM Trettene ADS Andrade LGMD Popim RC. Benefits of spirituality and/or religiosity in patients with chronic kidney disease: an integrative review. Rev Bras Enferm. (2019) 72:541–51. doi: 10.1590/0034-7167-2018-0051, PMID: 31017220

[21] Loureiro ACT de Rezende Coelho MC Coutinho FB Borges LH Lucchetti G. The influence of spirituality and religiousness on suicide risk and mental health of patients undergoing hemodialysis. Compr Psychiatry. (2018) 80:39–45. doi: 10.1016/j.comppsych.2017.08.004, PMID: 28972917

[22] Santos PR Capote Júnior JRFG Cavalcante Filho JRM Ferreira TP Dos Santos Filho JNG da Silva Oliveira S. Religious coping methods predict depression and quality of life among end-stage renal disease patients undergoing hemodialysis: a cross-sectional study. BMC Nephrol. (2017) 18:197. doi: 10.1186/s12882-017-0619-1, PMID: 28623903 PMC5474022

[23] Torres I Sippy R Bardosh KL Bhargava R Lotto-Batista M Goldsmith A. Chronic kidney disease in Ecuador: an epidemiological and health system analysis of an emerging public health crisis. PLoS One. (2022) 17:e0265395. doi: 10.1371/journal.pone.0265395, PMID: 35294504 PMC8926192

[24] Kidney Disease: Improving Global Outcomes (KDIGO) Blood Pressure Work Group. KDIGO 2021 Clinical Practice Guideline for the Management of Blood Pressure in Chronic Kidney Disease. Kidney Int. (2021) 99:S1–S87. doi: 10.1016/j.kint.2020.11.003 33637192

[25] Mezzadra JM Simkin A. Validación de la escala abreviada de afrontamiento religioso Brief-RCOPE en el contexto argentino en estudiantes de confesión católica. Revista Evaluar. (2017) 17:18–28. Recuperado de. Available at: https://revistas.unc.edu.ar/index.php/revaluar

[26] Valderrama Rios MC Sánchez R Sanabria M. Traducción y adaptación transcultural del instrumento Kidney Disease Questionnaire (KDQ) para la evaluación de calidad de vida en pacientes con enfermedad renal crónica en Colombia. Rev Colomb Nefrol. (2023) 10:687. doi: 10.22265/acnef.10.1.687

[27] Lucchetti G Almeida LG Granero AL. Spirituality for dialysis patients: should the nephrologist address? J Bras Nefrol. (2010) 32:126–30. doi: 10.1590/S0101-28002010000100020 PMID: 21448531

[28] Yıldırım Üşenmez T Demir Dikmen R. The effect of religious attitude on death anxiety among patients undergoing hemodialysis treatment: a sample from Turkey. J Relig Health. (2024) 63:2794–805. doi: 10.1007/s10943-024-02042-3, PMID: 38619688 PMC11319400

[29] Ramirez SP Macêdo DS Sales PMG Figueiredo SM Daher EF Araújo SM. The relationship between religious coping, psychological distress and quality of life in hemodialysis patients. J Psychosom Res. (2012) 72:129–35. doi: 10.1016/j.jpsychores.2011.11.012, PMID: 22281454

[30] Saffari M Pakpour AH Naderi MK Koenig HG Baldacchino DR Piper CN. Spiritual coping, religiosity and quality of life: a study on Muslim patients undergoing haemodialysis. Nephrology. (Carlton). (2013) 18:269–75. doi: 10.1111/nep.12041 23432815

[31] Vitorino LM Soares RDCES Santos AEO Lucchetti ALG Cruz JP Cortez PJO. Dos caras de la misma moneda: El impacto positivo y negativo del afrontamiento religioso espiritual en la calidad de vida y la depresión en pacientes en diálisis. J Holist Nurs. (2018) 36:332–40. doi: 10.1177/0898010117725429, PMID: 28836475

[32] Pilger C Caldeira S Rodrigues RAP Carvalho ECD Kusumota L. Spiritual well-being, religious/spiritual coping and quality of life among the elderly undergoing hemodialysis: a correlational study. J Relig Spiritual Aging. (2021) 33:2–15. doi: 10.1080/15528030.2020.1824848

[33] Vélez Jiménez D Mendoza Hernández J. Reconfiguración de la religiosidad del joven en la sociedad contemporánea. Sophia. (2020) 29:183–207. doi: 10.17163/soph.n29.2020.06

[34] Méndez NA Suazo SV Campo VR Ortiz JPH (2023). Calidad de vida en personas con tratamiento de hemodiálisis en Ecuador. En SciELO Preprints.

[35] Capote Leyva E Argundin Selier R Mora Gonzales SR Capote Pereira L Rupale IL Moret Hernandez Y. Assessment of health-related quality of life in patients on periodic haemodialysis using the KDQOL-SFTM. Medisur. (2015) 13:508–16. Available at: http://www.medisur.sld.cu/index.php/medisur/article/view/2789

[36] Puchalski CM Sbrana A Ferrell B Jafari N King S Balboni T. Interprofessional spiritual care in oncology: a literature review. ESMO Open. (2019) 4:e000465. doi: 10.1136/esmoopen-2018-000465, PMID: 30962955 PMC6435249

